# Inbreeding Depression and Purging for Meat Performance Traits in German Sheep Breeds

**DOI:** 10.3390/ani13223547

**Published:** 2023-11-17

**Authors:** Cathrin Justinski, Jens Wilkens, Ottmar Distl

**Affiliations:** 1 Institute of Animal Breeding and Genetics, University of Veterinary Medicine Hannover (Foundation), 30559 Hannover, Germany; cathrin.justinski@tiho-hannover.de; 2 VIT—Vereinigte Informationssysteme Tierhaltung w.V., Heinrich-Schröder-Weg 1, 27283 Verden, Germany; jens.wilkens@vit.de

**Keywords:** inbreeding depression, sheep breeds, production traits, meat performance, daily weight gain, purging

## Abstract

**Simple Summary:**

In meat sheep and sheep bred for other purposes, meat quality is one of the factors evaluated in a breeding programme. In this study, we included 25 sheep breeds from Germany with a sufficient amount of performance and pedigree data. The heritability, genetic and residual correlations were estimated for daily weight gain, meatiness score and ultrasound measurements for the muscle and fat thickness of the sheep breeds. We employed animal models to analyse inbreeding depression by estimating linear slopes of inbreeding coefficients on meat performance traits. For daily weight gain, inbreeding depression was significant for all sheep breeds. When considering single breeds, significant inbreeding depression was only found in two breeds. The meatiness scores did not show significant inbreeding depression across breeds, nor in single breeds, but only in one breed. The ultrasound measurements for muscle and fat thickness were not significant for inbreeding depression either across or within breeds. Purging effects did not prove to be significant either across or within breeds. Our data show that inbreeding depression and purging can be present in traits under strong selection.

**Abstract:**

This study provides estimates on genetic parameters, inbreeding depression and purging for meat performance measures from 25 German sheep breeds. All German meat, merino sheep breeds and breeds of other breeding directions with a sufficient number of pedigree and performance data were included in this study. Phenotypic traits retrieved from the national database OviCap were evaluated: daily weight gain, meatiness score and ultrasound measurements for muscle and fat thickness. We employed animal models to estimate heritability, variance and covariance components for these meat performance traits as well as inbreeding depression and purging. The heritabilities, on average, reached estimates of 0.55, 0.34, 0.53 and 0.61 for daily weight gain, meatiness score and ultrasound measurements for muscle and fat thickness, respectively. We estimated the linear regression slopes for the individual rate of inbreeding, new and ancestral inbreeding, as well as the inbreeding coefficient and its interaction with the inbreeding coefficient of Ballou, employing animal models with non-genetic effects and the additive genetic effect of the animal. Across all breeds, inbreeding was only significant for daily weight gain, whereas for all other traits, estimates were not significant. Within sheep breeds, we found significant inbreeding depression for daily weight gain in German Mutton Merino and German Blackheaded Mutton as well as for the meatiness score in German Whiteheaded Mutton. Significant effects for purging, based on ancestral inbreeding and the interaction effect of the classical inbreeding coefficient with the inbreeding coefficient of Ballou, were not obvious either across or within any sheep breed. A 1% increase in inbreeding significantly decreased the phenotypic trait median of daily weight gain across all sheep breeds by 0.50% and 0.70% of phenotypic and genetic standard deviation, respectively. Purging effects due to ancestral inbreeding were not significant in any breed or across breeds. The results of this study may indicate that inbreeding depression may be more harmful in traits under stronger selection than in traits that exert low selection pressure. The results of this study demonstrate the different effects that result in meat performance traits due to inbreeding. With increasing rates of inbreeding and critical effective population sizes, selection intensity for breeding objectives has to be critically reviewed for each sheep breed. Inbreeding depression and purging should be evaluated in order to prevent a decrease in trait means due to inbreeding and to determine whether detrimental alleles are eliminated.

## 1. Introduction

Sheep breeds in Germany have adapted to a large variety of ecosystems, and many specialised breeding types have become established over the decades of selective breeding [1]. The diversity of German sheep breeds also reflects different breeding objectives caused by climatic and environmental conditions. As there was a change in industrialisation, there was also a change in the selection criteria of sheep breeding [2]. For example, daily weight gain and meat quality became more and more important and were forced to complement or even replace the previously dominant criteria of wool quality and external appearance. As also proven in our first study, almost all of the studied breeds suffered different genetic bottlenecks and more or less severe losses of their genetic diversity during their evolutionary history. Thus, the question naturally arises as to how strongly additional selection criteria, besides genetic drift and unequal founder contributions, affect genetic resources and to what extent the problem of inbreeding depression has manifested itself in different breeds through breeding interventions.

Comparative studies by Doekes [3] and Leroy [4] on inbreeding depression in many domestic animal species showed the need for detailed research in this area (Table S1). On closer examination of single breeds, their origin and breeding history, it became clear that there are major differences that must absolutely be taken into account during evaluations. For as just as different and diverse as the characteristics and phenotypes of the internationally compared sheep breeds are, so are the breeding and selection criteria of their countries of origin [5]. Even though there are existing studies on the correlation of meat-performance-associated traits and inbreeding depression, none of the work is sufficient because there are no mandatory globally comparable selection criteria and thus no direct comparison of the available results. For example, it is recognized and proven that there are breed-dependent differences in meat traits [6], some of which have also been elicited using comparable methods [7], but there is no work that compares the results collected using the same methodology within such a large breed range, as is the case in this work.

The largest number of studies on inbreeding depression in sheep populations concern the trait complex of production and growth and include the traits of daily weight gain, body weight at different stages of development and weaning weight, as well as, in a number of studies, birth weight. The breeds (Horro [8], Elsenburg Dormer [9], Santa Ines [10,11], Moghani [12,13] Iran Black [14,15], Lori [16] and Shal [17]) were investigated with regard to the parameter body weight comparable to breeds in this study and showed significant evidence of negative influences on weight caused by inbreeding depression. Losses in daily weight gain caused by inbreeding have also been demonstrated in studies on various breeds. In addition to the Scottish breeds, Scottish Blackface, Cheviot, and Welsh Mountain studied by Wiener [18], this phenomenon has also been shown in the Elsenburg Dormer [19], Iran Black [15] and Santa Ines [11]. Norberg and Soerensen also found significant reductions in phenotypic means due to inbreeding for all traits studied in the Oxford Down, Shropshire and Texel breeds [20].

In addition to the general breeding goal of avoiding inbreeding depression to preserve genetic variation in captive populations, purging assumes that intensive selection can lead to the elimination of deleterious alleles from a population, thus counteracting inbreeding depression. By ridding the population of this genetic load, the fitness of the population should therefore not be affected in the long term [21]. According to the theory, after a few generations of inbreeding, the best-performing inbred individuals will survive and thus achieve a level of performance comparable to or higher than that of less or non-inbred individuals, while carriers of deleterious alleles will die or fail to reproduce, thus eliminating their deleterious alleles from the population [22,23]. However, recent studies suggest that selection may be able to counterbalance the adverse effects, particularly of milder inbreeding, and that inbreeding may be an effective tool to maintain genetic diversity between different lines [24]. Purging can be considered not only as a phenomenon, but also as an important tool for the conservation of genetic resources when used selectively in breeding programmes to detect and eliminate recessive deleterious mutations through natural selection [25].

The aim of this work was to analyse the effects of inbreeding for meat performance traits in all sheep breeds in Germany, participating in progeny testing of lambs within the breeding programmes.

## 2. Materials and Methods

The data for this study were retrieved from OviCap, the nationwide database for sheep and goats in Germany, which is managed by vit/Verden, Germany. As a follow-up study to previous work [1], 25 breeds that were already been investigated for genetic diversity are included here (Table S2). For sheep breeds in Germany, a progeny test is offered for meat performance by evaluating the lambs. The meat performance test for lambs is not compulsory for all breeds (Table S3). Depending on the state, the meat performance test can be carried out as a station or field test. The parameters recorded are daily weight gain in grams, meatiness score (score 1–9, with 9 being optimal), and ultrasound measurements of muscle and fat thickness in millimetres (Figure S1). Recordings are carried out by trained staff from the Sheep Breeders’ Associations and recorded in the herd book and OviCap as a separate performance test for the lambs.

The field test is organised by the respective breeding association for the lambs whose parents are registered in the herd book. The field test takes place when the majority of the flock has reached the commercial fattening weight typical of the breed. The live weight on the day of measurement should be within a range of ±10 kg of the target final weight. The age of the lambs at the time of field performance testing must be between 60 and 210 days. For males and females, at least daily weight gain in grams (g) is recorded. The standard birth weight for the breed in question is subtracted from the test weight and then divided by the age in days on the day of the test. Alternatively, the breeder can enter the actual birth weight in OviCap. In particular, meat breeds are scored for conformation and muscle and fat thickness by a breed association representative.

To assess meatiness, the breast, shoulder, back and club were scored and weighted according to their economic importance with values from 1–9.

The ultrasound-aided measurements readings were conducted caudal to the 13th rib on the right side of the body. The measurements were recorded in millimetres with one decimal place. The ultrasound muscle thickness was recorded as the largest vertical cross section of the muscle including the muscle fascia (Figure S1). The ultrasound fat thickness is the extension of the vertical line that captures the largest vertical cross-section of the muscle and includes the overlying fat layer, including the skin.

During the measurement, the lambs were fixed in a special rack so that their legs did not touch the ground and the back musculature was relaxed. The wool, which may impair the ultrasound measurement, is parted straight at the measurement site with a thin, pointed object and oil or ultrasound gel is applied to the skin as a contact medium. In order to rule out any measurement errors due to the contact medium, the ambient temperature should be at least 8 °C.

The test location, lamb identification number, test date, and test weight were recorded in OviCap. Weight class correction is applied for the trait ultrasound muscle thickness and ultrasound fat thickness within breeds. According to the live weight for ultrasound measurement, 10 approximately equal classes were formed within each breed (Table S4).

The inbreeding coefficients according to Meuwissen and Luo [26], Ballou [27], Kalinowski et al. [28] and Baumung et al. [29] detected in our previous study [1] using PEDIG [30] and GRAIN, version 2.2 [31] were used for this study (Table S2).

The heritabilities, residual and genetic correlations and (co-)variances of daily weight gain, meatiness score, ultrasound muscle thickness and ultrasound fat thickness were estimated separately for each sheep breed. Sufficient data were available for daily weight gain for 25 sheep breeds and for the other traits for 12 sheep breeds.

The following linear multivariate animal model, parameterised according to the models used in the routine evaluation of breeding values by Vit/Verden, was used to estimate variances and covariances.

Model 1 for daily weight gain or meatiness score and model 2 for ultrasound muscle and ultrasound fat thickness are as follows:

Y_ijkl_ = µ + LOC-YEAR_i_ + SEX-MULTIPLES_j_ + AGE_k_ + animal_l_ + e_ijkl_, (1)

Y_ijkl_ = µ + LOC-YEAR_i_ + SEX-MULTIPLES_j_ + WEIGHT_k_ + animal_l_ + e_ijkl_, (2)

where Y_ijkl_ = daily weight gain, meatiness score, ultrasound muscle or fat thickness measurement. LOC-YEAR_i_ is the ith location by year effect for each breed with different numbers of levels for each breed; SEX-MULTIPLES_j_ is the jth class of the sex of the animal by number of animals born per lambing for j = 1–2 (sex) by 1–4 (up to four multiples, depending on sheep breed); AGE_k_ = age at testing in days by six classes for k = 1 (60–129), 2 (130–159), 3 (160–189), 4 (190–365), 5 (360–729) and 6 (730–1095); WEIGHT_k_ = weight divided into 10 classes within each breed k = 1 (<−1.28), 2 (−1.28 to −0.84), 3 (−0.84 to −0.52), 4 (−0.52 to −0.25), 5 (−0.25 to 0), 6 (0 to 0.25), 7 (0.25 to 0.52), 8 (0.52 to 0.84) 9 (0.84 to 1.28), 10 (>1.28); animal_l_ = random animal effect, and e = random error term.

The effects of the different inbreeding coefficients were analysed by extending models 1 and 2 with linear regressions (b_1_ to b_5_) on the respective inbreeding coefficients. We used three differently parameterised models to analyse the effects of inbreeding coefficients. Model 3 considered the individual rate of inbreeding (ΔF_i_).

Model 3:

Y_ijklm_ = µ + LOC-YEAR_i_ + SEX-MULTIPLES_j_ + AGE_k_ or WEIGHT_k_ + b_1_ΔF_il_ + animal_m_ + e_ijklm_

In model 4, the effect of the ancestral inbreeding coefficient (F_a_Kal_) and the new inbreeding coefficient (F_a_New_) according to Kalinowski et al. (2000) [28] were analysed simultaneously.

Model 4:

Y_ijklmn_ = LOC-YEAR_i_ + SEX-MULTIPLES_j_ + AGE_k_ or WEIGHT_k_ + b_2_F_a_Newl_ + b_3_F_a_Kalm_ + animal_n_ + e_ijklmn_

In model 5, the classical inbreeding coefficient (F) and the interaction of F_a_Bal_ with F, as proposed by Ballou (1997) [27], were considered simultaneously, since the probability that an individual is identical by descent (IBD) is not itself taken into account by F_a_Bal_.

Model 5:

Y_ijklmn_ = LOC-YEAR_i_ + SEX-MULTIPLES_j_ + AGE_k_ or WEIGHT_k_ + b_4_F_l_ + b_5_F×F_a_Balm_ + animal_n_ + e_ijklmn_

The variance components were estimated for daily weight gain or simultaneously for all four traits with models 1 and 2 using VCE 6.0.2 [32,33]. The variances and covariances were estimated with REML (restricted maximum likelihood) using analytical gradients. The size of the pedigree and trait data sets are given in Table 1. For models 2–4, we used estimated additive genetic and residual variances to estimate effects for linear regressions using PEST, version 4.2.6. Further statistical analyses were performed using SAS, version 9.4 (Statistical Analysis System, Cary, NC, USA, 2023).

## 3. Results

Table 1 shows the 25 breeds examined in this study, including the number of animals with pedigree data, the number of complete equivalent generations (GEs) and the number of daily weight gain records. For 12/25 sheep breeds, a sufficient number of lambs were tested for the meatiness score, ultrasound muscle thickness, and ultrasound fat thickness (Table 2).

### 3.1. Estimated Heritabilities, Residual and Genetic Correlations and (co-)Variances

The mean (median) heritability for daily weight gain was 0.54 (0.53) on average across all breeds (Table 1 and Table S4). The first and third quartiles were 0.49 and 0.62 and included 8/25 and 19/25 breeds, respectively. The breeds BLS and WGH showed the highest estimates with 0.73 ± 0.073 and 0.75 ± 0.09, respectively, whereas DOS and TEX showed the lowest estimates with 0.34 ± 0.09 and 0.36 ± 0.02, respectively.

For the meatiness score, the mean (median) heritability was 0.31 (0.30) on average for all breeds (Table 2 and Table S4). The first and third quartiles were 0.275 and 0.36 and included 3/12 and 8/12 breeds, respectively. The breed CHA showed the highest estimate with 0.42 ± 0.10 and MLW the lowest estimate with 0.21 ± 0.05.

The mean (median) heritability for ultrasound muscle thickness was 0.53 (0.55) on average across breeds (Table 2 and Table S4). The first and third quartiles were 0.425 and 0.645 and included 4/12 and 9/12 breeds, respectively. The breed MLS showed the highest estimate with 0.87 ± 0.02 and MLW the lowest estimate with 0.21 ± 0.05.

The mean (median) heritability for ultrasound fat thickness was 0.61 (0.61) on average for all breeds (Table 2 and Table S4). The first and third quartiles were 0.47 and 0.75 and included 4/12 and 9/12 breeds, respectively. The breed MLS showed the highest estimate with 0.86 ± 0.02 and SUF the lowest with 0.38 ± 0.02.

### 3.2. Inbreeding Depression for Daily Weight Gain

#### 3.2.1. Individual Rate of Inbreeding

The animal model regression coefficients for daily weight gain showed positive results for 7/25 breeds (BLS, CHA, OMS, BRI, IDF, SUF, DOS) but were not significant. The estimates for the remaining 18/25 breeds were negative, but only two estimates for the breeds MFS and SKF (−377.216 ± 163.95, −386.916 ± 144.52) were significant (Table 3 and Table S5a). The strongest negative result was obtained for WGH, but the estimate had a very large standard error and was therefore not considered meaningful. For three breeds (BDC, GGH, RHO), the estimates were smaller than −300, but with large standard errors and thus not significantly different to zero. The mean rates of inbreeding for all breeds were significantly negative, except for ΔF_i_/trait mean (Table 4 and Table 5).

#### 3.2.2. Ancestral and New Inbreeding According to Kalinowski

For F_a_Kal_, 2/25 breeds (CHA, OMS) had significantly negative values (−1119.826 ± 428.68, −502.833 ± 219.95) and 12/25 breeds had positive regression coefficients (BBS, BDC, BLS, DOS, IDF, MFS, MLS, MLW, SKF, WAD, WGH, WHH). F_a_New_, on the other hand, showed 8/25 breeds (BLS, BRI, CHA, COF, GGH, KST, OMS, WBS) with positive regression coefficients with this trait, two of which were significant (CHA, OMS) with values 201.905 ± 104.99 and 163.334 ± 77.05. Of the remaining negative values, two results (−101.943 ± 34.62, −95.315 ± 45.49) were also significant, namely for the breeds SKF and MFS (Table S5b). However, the overall means were not significant (Table 4 and Table 5).

#### 3.2.3. Interaction of F×F_a_Bal_ and F

A positive regression between F and daily weight gain was found for 6/25 breeds (BLS, BRI, CHA, OMS, IDF, WAD), but no significance could be demonstrated. The breeds MFS, SKF and MLS showed significantly negative values (−69.853 ± 27.93, −62.586 ± 22.76, −42.673 ± 21.33) (Table S5c). For the examined interaction F×F_a_Bal_, which is indicative of purging, a negative result was estimated for 10/25 breeds (AST, BRI, CHA, COF, KST, LES, OMS, TEX, WBS, WKF); only for the breed CHA (−2919.469 ± 1067.53) was this value significant (Table S5c). We were not able to show significant averages between breeds (Table 3 and Table 4).

### 3.3. Inbreeding Depression for the Meatiness Score

The overall averages for all inbreeding coefficients were not significant (Table 4 and Table 5).

#### 3.3.1. Individual Rate of Inbreeding

The regression for this trait showed a significant negative value for the breed WKF (−5.723 ± 2.48) (Table S6a).

#### 3.3.2. Ancestral and New Inbreeding According to Kalinowski

For F_a_Kal_, three breeds (DOS, MLW, MLS) were estimated with positive regression coefficients, but no significance could be demonstrated. Also for F_a_New_, no significance was found either for the 8/12 breeds (CHA, DOS, MFS, MLS, MLW, SKF, TEX, WKF) with negative regression coefficients or for the 4/12 (BDC, IDF, LES, SUF) with positive regression coefficients (Table S6b).

#### 3.3.3. Interaction of F×F_a_Bal_ and F

For the regression on the meatiness score, significant negative results (−1.145 ± 0.5, −0.984 ± 0.44) were obtained for F for 2/12 breeds (WKF, MFS). For the interaction of F×F_a_Bal_, 4/12 breeds (DOS, TEX, MLS, SKF) showed positive values, but none of the breeds reached the significance threshold (Table S6c).

### 3.4. Inbreeding Depression for the Ultrasound Muscle Thickness

Overall averages for all inbreeding coefficients were not significant (Table 3 and Table 4).

#### 3.4.1. Individual Rate of Inbreeding

No significant results were obtained for ultrasound muscle thickness, and 3/12 breeds (MLS, MLW, DOS) had a positive result (Table S7a).

#### 3.4.2. Ancestral and New Inbreeding According to Kalinowski

F_a_Kal_ also showed no significance; here 4/12 breeds (MLW, WKF, DOS, TEX) had positive regressions with ultrasound muscle thickness. Similarly for F_a_New_, 4/12 breeds (LES, DOS, MLS, SKF) showed positive regressions, but without detectable significance (Table S7b).

#### 3.4.3. Interaction of F×F_a_Bal_ and F

Ultrasound muscle thickness showed a positive regression for F in 2/12 breeds (MLW, DOS), but none of the values were significant. The interaction between F and F_a_Bal_ gave positive estimates in 6/12 breeds (MLW, BDC, WKF, DOS, IDF, TEX), but again no significance could be demonstrated (Table S7c).

### 3.5. Inbreeding Depression for the Ultrasound Fat Thickness

Overall averages for all inbreeding coefficients are given in Table 4 and Table 5, but the estimates were not significant.

#### 3.5.1. Individual Rate of Inbreeding

The situation for ultrasound fat thickness was similar to that for ultrasound muscle thickness, with 3/12 breeds (WKF, IDF, MLW) showing positive regression coefficients, but no significance (Table S8a).

#### 3.5.2. Ancestral and New Inbreeding According to Kalinowski

F_a_Kal_ showed negative regressions for 4/12 breeds (CHA, MFS, LES, SUF), while the remaining 8/12 showed positive values, but none were significant. Additionally, for F_a_New_, no significance could be demonstrated for the 4/12 breeds (DOS, CHA, MLS, SUF) with positive estimates and the 8/12 breeds with negative estimates (Table S8b).

#### 3.5.3. Interaction of F×F_a_Bal_ and F

Estimates of F showed positive regressions in 6/12 breeds (MLW, DOS, CHA, MLS, TEX, SUF), but neither for these nor the negative coefficients were significant. Also, for the interaction of F with F_a_Bal_, no significance could be demonstrated. In 3/12 breeds (MLW, IDF, WKF), positive values were estimated.

### 3.6. Inbreeding Depression by Breeding Directions

When comparing the regression coefficients in the different breeding groups for daily weight gain, none of the estimates by breeding group were significant for any of the inbreeding coefficients (Table S5d). On average, the heath sheep had a more pronounced inbreeding depression (−648.192 ± 173.112) than the meat, country and mountain-stone sheep in the individual rate of inbreeding.

None of the breeding directions showed significant values for the trait meatiness score for any of the inbreeding coefficients (Table S6d). No significant results could be obtained for any of the inbreeding coefficients for ultrasound muscle and fat thickness for averages of breeding directions (Table S7d).

## 4. Discussion

Various models exist to estimate inbreeding depression in livestock, the standard procedure being a regression of individual performance on the individual pedigree inbreeding coefficient [34]. In this study, the individual inbreeding rate ∆F_i_ developed by Gutierrez et al. [32] was used instead of the pedigree coefficient to account for differences in pedigree depth. In practice, several studies in livestock species considering both linear and non-linear models [35,36] have concluded that linear models fit well at low to moderate levels of inbreeding (below 10–20%). In wild species, selection occurs mainly for fitness traits, whereas in livestock, selection also occurs for production-related traits. These traits are affected by inbreeding due to intensive selection, whereas in nature, they would not show inbreeding depression. Traits with high heritability are be expected to have higher accuracy in estimating inbreeding depression. The results of Leroy’s meta-analysis show that any type of selected trait can be affected by inbreeding depression in livestock [4]. Heritability estimates have been reported in available comparable studies, for which the values are available for comparable parameters in the Rambouillet sheep [37]. Estimates of heritabilities and variance components for traits comparable to our work, such as average daily weight gain, are available for the Iranian Sangsari breed [38]. However, the estimated heritability in this study (0.01) was significantly lower than in our breeds. Safari et al. also undertook a comprehensive study of heritability and variance estimation, comparing different traits in sheep from different uses and breeding directions [39]. Comparative estimates of variances, heritabilities and potential maternal effects on different traits were conducted using different models in the Afrino sheep in order to establish a suitable estimation model [40]. Further variance components and heritabilities of growth traits have been estimated for Elsenburg Dormer sheep [41] and Lori-Bakhtiari sheep [42].

The regression coefficients estimated within the animal model for the association of daily weight gain with individual inbreeding rate were negative in most breeds, with two of the breeds showing significantly negative results. The pattern of distribution of estimates between breeds shows that, in particular, breeds selected for this trait have more negative regression coefficients and thus increased inbreeding depression. In addition to various hypotheses, for example in the work of Leroy [4], forced breeding selection for one or more traits within a certain trait complex seems to be an increased predisposition for the development of inbreeding depression. The results of this study support the latter hypothesis. For example, breeds that have not been specifically selected for meat performance appear to be less affected by inbreeding depression than those whose breeding programmes include selection for meat performance as a mandatory requirement. In comparable studies, the authors also concluded that inbreeding depression had a greater effect on production-related traits [43]. An often-debated question is whether the effect of inbreeding depression on life history and/or fitness traits makes a difference, and if so, what difference. For example, a meta-analysis of animal populations found no particular difference between life-history, morphological, and physiological trait types in the correlation between traits and molecular heterozygosity [44]. DeRose and Roff (1999) interpreted this difference by suggesting that there is less directional dominance in morphological traits than in fitness traits [43]. Taking into account the dominance effect d for a given locus, inbreeding depression can be expressed as 2dpqF [45]. Our results support this hypothesis. Therefore, inbreeding is detrimental if the dominance effect over the loci is positive, which is the case in selection, because fixation is faster at loci with negative d. These traits would be all the more affected by inbreeding because they are intensively selected, whereas in nature they would not show inbreeding depression. For traits with high heritability, we can also expect higher accuracy in estimating inbreeding depression [43].

Estimates of daily weight gain in 2/25 breeds (MFS, SKF) showed a significant negative regression (−377.216 ± 163.95, −386.916 ± 144.52 caused by new inbreeding in both breeds. As recent inbreeding is known to have more detrimental effect on sheep than ancestral inbreeding, regressions of ancestral and recent inbreeding according to Kalinowski were considered with special attention to possible inbreeding adjustment. As F_a_Kal_ was significantly negative in only 2/25 (CHA, OMS) and almost half (12/25) of all breeds examined showed a positive regression for daily weight gain, but without a demonstrable significance, we cannot exclude a purging effect for this trait. The interaction of F and F_a_Bal_ and thus a possible purging effect on daily weight gain in the Charollais breed was unlikely (−2919.469 ± 1067.53). The results for the meatiness score were indifferent; a significant negative regression and therefore inbreeding depression for this trait could only be shown in the breed WKF (−5.723 ± 2.48). For the traits meatiness score (3/12), ultrasound muscle thickness (4/12) and ultrasound fat thickness (8/12), some breeds always showed positive but not significant regressions, and two of the breeds examined (MLW, DOS) were always among them. Almost the same breeds also showed positive regressions for the interaction of F with F_a_Bal_, but again no significance could be found. Therefore, a purging effect in the breeds examined in this study is not proven for the traits evaluated. However, it cannot be completely excluded. A significant negative regression for daily weight gain (−146.9863 ± 67.8317) was found for all the breeds studied, and this was confirmed in the individual breeding lines.

Even if the present results show some evidence of inbreeding depression to varying degrees, the phenomenon could not be detected significantly in a large number of breeds. However, breeds with significant inbreeding depression, such as MFS and SKF for daily weight gain or WKF for meatiness score, have been explicitly bred for high meat production and thus for the parameters studied here, and these breeds have been selected for this over generations. The question then arises as to what effect this previous selection pressure had on the breed in question, or even on the population as a whole. For MFS and SKF, the current inbreeding depression could be proved significantly by the new inbreeding according to Kalinowski, whereas for WKF this could not be demonstrated due to a lack of significance; however, due to a more negative b-value for F_a_Kal_ compared to F_a_New_, it can be assumed that the cause here is strengthened by the already existing ancestral inbreeding.

The comparative meta-analysis by Doekes [3], particularly in relation to the sheep breeds already studied, led us to expect similarly strong effects of inbreeding depression in the German breeds due to the selection pressure. To compare the present results with previous reports, the estimates of the regression coefficients were scaled to the respective phenotypic (b_σp_) and additive genetic standard deviations (b_σa_) of the traits using the variance components from models 1 and 2. For daily weight gain, meatiness score, ultrasound muscle and fat thickness, the median per 1% increase in F scaled to b_σp_ was −0.50% (CI25–75%: −1.08 to −0.23, skewness = −0.75, kurtosis = 2.63), −0.56% (CI25–75%: −1.41 to 1.05, skewness = 0.08, kurtosis = −0.93), −0.50% (CI25–75%: −1.24 to −0.20, skewness = 0.74, kurtosis = 0.45), and −0.31% (CI25–75%: −1.23 to 0.64, skewness = 2.15, kurtosis = 5.20), respectively. The corresponding medians of the same traits per 1% increase in F scaled to b_σa_ were −0.70% (CI25–75%: −1.61 to −0.32, skewness = −0.39, kurtosis = 1.29), −1.00% (CI25–75%: −2.94 to −1.80, skewness = 0.24, kurtosis = −1.19), −0.75% (CI25–75%: −1.80 to −0.24, skewness = 1.65, kurtosis = 3.40), and −0.33% (CI25–75%: −1.57 to 0.81, skewness = 2.43, kurtosis = 6.69), respectively. These estimates for inbreeding depression were considered similarly deleterious to the overall median of −0.52% (CI25–75%: −1.43 to −0.04, skewness = 4.26, kurtosis = 47.36) for b_σp_, calculated for F from the Doekes meta-analysis for sheep, and the median across species and traits of −0.59% [3]. Using only the trait categories of weight/growth and reproduction for the sheep data from the meta-analysis [3], we found estimates of −0.73% (CI25–75%: −1.38 to −0.27, skewness = −2.66, kurtosis = 6.81) and −0.38 (CI25–75%: −1.99 to 0.08, skewness = 3.46, kurtosis = 20.43), respectively. However, this was only partially confirmed when looking at individual sheep breeds. The sheep breeds described by Doekes, as well as the selection criteria according to which these breeds were bred and thus developed, differ significantly from the parameters investigated in this study, as the scheme used in Germany and carried out here by the breeding associations, is unique and the results are therefore not directly comparable with other studies [5]. Nevertheless, results were obtained in a similar range for the different breeds.

## 5. Conclusions

Inbreeding depression due to individual rate of inbreeding and the classical inbreeding coefficient across all 25 sheep breeds was evident for daily weight gain. The effect of inbreeding depression, expressed as a 1% increase in the classical inbreeding coefficient per phenotypic standard deviation of the trait, was most important for daily weight gain and least important for ultrasound fat thickness. Inbreeding depression was more pronounced when expressed per additive genetic standard deviation of the trait. The individual rate of inbreeding proved to be a more sensitive measurement for the evaluation of inbreeding depression and purging. The beneficial effects of alleles that were identical by descent in ancestral generations appear to play a role in daily weight gain and ultrasound fat thickness in sheep breeding. However, significant effects could not be demonstrated for single breeds or across breeds. Efforts to maintain genetic diversity in sheep should also consider the intensity of selection for breeding objective traits to reduce the effects of inbreeding depression. The results of the present study are useful for the evaluation of sheep breeding programmes and selection intensity for meat performance, especially for endangered sheep breeds in Germany.

## Figures and Tables

**Table 1:** Sheep breeds analysed, grouped into breeding directions (BDs) including number of animals in the pedigree dataset (N_ped_), generation equivalent (GE), and number of animals (n) with records for daily weight gain and mean values with their standard deviations (SD) and heritability estimates with their standard errors (SE).

Code	BD	Breed	N_ped_	GE	Daily Weight Gain (g/Day)		
					n	Mean ± SD	h^2^ ± SE
AST	MON	Alpine Steinschaf	10,420	4.94	1047	233.51 ± 67.01	0.41 ± 0.05
BBS	MON	Brown Mountain	22,961	6.22	1269	286.86 ± 63.75	0.51 ± 0.04
BDC	EXO	Berrichon du Cher	4680	3.69	454	359.23 ± 75.24	0.66 ± 0.06
BLS	CON	Bentheim	46,173	8.91	442	262.79 ± 63.97	0.73 ± 0.07
BRI	CON	Carinthian	10,669	4.26	800	267.93 ± 70.83	0.46 ± 0.08
CHA	MEA	Charollais	11,237	3.25	380	339.66 ± 69.03	0.62 ± 0.08
COF	CON	Coburg	70,156	8.15	1544	267.4 ± 76.39	0.66 ± 0.01
DOS	MEA	Dorper	36,057	6.13	359	272.97 ± 54.84	0.34 ± 0.09
GGH	HEA	German Grey Heath	69,369	8.57	919	210.72 ± 42.99	0.61 ± 0.06
IDF	MEA	Ile-de-France	14,021	4.07	673	343.39 ± 89.43	0.56 ± 0.04
KST	MON	Krainer Steinschaf	9671	5.54	1023	243.06 ± 71.11	0.52 ± 0.04
LES	CON	Leine	42,949	8.70	1617	281.12 ± 78.79	0.62 ± 0.03
MFS	MER	German Mutton Merino	132,413	7.33	3387	312.51 ± 70.18	0.51 ± 0.02
MLS	MER	German Merino	204,494	8.52	6746	388.41 ± 69.79	0.53 ± 0.02
MLW	MER	Merino Longwool	61,216	6.66	569	387.61 ± 46.16	0.55 ± 0.05
OMS	MIL	East Friesian	71,159	9.06	1447	351.62 ± 78.18	0.63 ± 0.03
RHO	CON	Rhön	78,095	7.19	508	273.93 ± 65.49	0.72 ± 0.07
SKF	MEA	German Blackhead Mutton	128,839	7.91	9224	399.17 ± 91.19	0.39 ± 0.01
SUF	MEA	Suffolk	68,136	5.27	5759	389.82 ± 88.96	0.40 ± 0.02
TEX	MEA	Texel	58,223	5.79	4251	371.90 ± 75.77	0.36 ± 0.02
WAD	CON	Wald	17,172	5.77	673	212.33 ± 70.97	0.59 ± 0.06
WBS	MON	White Mountain	30,188	8.20	2119	303.42 ± 74.93	0.49 ± 0.03
WGH	HEA	German White Heath	18,158	8.27	377	188.47 ± 59.45	0.75 ± 0.09
WHH	HEA	White Polled Heath	41,306	9.94	709	189.44 ± 43.83	0.52 ± 0.06
WKF	MEA	German Whitehead Mutton	38,390	7.49	1442	344.26 ± 72.38	0.49 ± 0.03

Abbreviations for breeding directions: CON: country, EXO: exotic, HEA: heath, MEA: meat, MER: merino, MIL: milk, MON: mountain-stone.

**Table 2:** Meat performance traits with the number of sheep recorded (N), their mean values with their standard deviations (SD), and their heritabilities (h^2^) with their standard errors (SE) estimated for daily weight gain, meatiness score, ultrasound muscle thickness, and ultrasound fat thickness.

Breed	Meatiness Score (1–9)			Ultrasound Muscle Thickness (mm)			Ultrasound Fat Thickness (mm)		
	N	Mean ± SD	h^2^ ± SE	N	Mean ± SD	h^2^ ± SE	N	Mean ± SD	h^2^ ± SE
BDC	333	7.76 ± 0.53	0.37 ± 0.06	343	32.13 ± 3.96	0.61 ± 0.07	343	7.26 ± 1.83	0.71 ± 0.07
CHA	296	7.86 ± 0.74	0.42 ± 0.10	400	32.20 ± 5.53	0.54 ± 0.06	402	6.85 ± 2.60	0.68 ± 0.06
DOS	180	7.66 ± 0.64	0.26 ± 0.10	218	30.07 ± 3.59	0.66 ± 0.08	218	5.15 ± 1.38	0.79 ± 0.08
IDF	349	7.78 ± 0.72	0.41 ± 0.07	379	30.97 ± 3.97	0.63 ± 0.07	377	5.95 ± 1.61	0.47 ± 0.04
LES	384	7.49 ± 0.69	0.35 ± 0.05	249	27.52 ± 3.11	0.37 ± 0.06	249	5.92 ± 1.12	0.52 ± 0.05
MFS	2579	7.31 ± 0.85	0.28 ± 0.02	1998	27.99 ± 3.60	0.43 ± 0.02	2022	5.82 ± 1.48	0.56 ± 0.02
MLS	1178	7.72 ± 0.66	0.28 ± 0.03	1204	31.75 ± 6.54	0.87 ± 0.02	1205	7.13 ± 3.15	0.86 ± 0.02
MLW	262	7.56 ± 0.76	0.21 ± 0.05	197	30.18 ± 3.40	0.15 ± 0.05	197	5.75 ± 1.46	0.47 ± 0.05
SKF	6162	7.72 ± 0.62	0.31 ± 0.01	3598	29.93 ± 3.80	0.68 ± 0.01	3612	6.66 ± 1.73	0.82 ± 0.01
SUF	3766	7.80 ± 0.66	0.30 ± 0.02	4031	32.30 ± 4.90	0.55 ± 0.02	4035	6.89 ± 2.35	0.38 ± 0.02
TEX	3395	7.92 ± 0.57	0.27 ± 0.02	3945	32.65 ± 4.51	0.42 ± 0.02	3949	6.93 ± 2.04	0.45 ± 0.02
WKF	1018	7.63 ± 0.54	0.32 ± 0.05	1123	31.45 ± 3.95	0.46 ± 0.04	1127	8.01 ± 2.27	0.66 ± 0.03

**Table 3:** Animal model regression coefficients of the individual rate of inbreeding (∆F_i_) on the traits daily weight gain, meatiness score, ultrasound muscle, and ultrasound fat thickness with their corresponding standard errors (SE). Negative regression coefficients indicate inbreeding depression. Significant regression coefficients are in bold.

Breed	Daily Weight Gain	SE	Meatiness Score	SE	Ultrasound Muscle Thickness	SE	Ultrasound Fat Thickness	SE
AST	−219.376	176.184						
BBS	−158.9950	165.840						
BDC	−319.017	240.206	2.332	2.926	−15.580	16.799	−6.048	7.851
BLS	539.066	971.206						
BRI	139.508	164.613						
CHA	231.749	190.606	−2.839	2.335	−11.899	16.399	5.734	7.467
COF	−37.209	272.417						
DOS	3.339	362.405	6.766	6.501	20.845	32.880	19.039	13.786
GGH	−382.008	427.587						
IDF	22.494	115.652	−0.551	2.426	−5.107	6.291	−3.184	2.891
KST	−66.466	212.277						
LES	−69.414	228.218	7.896	7.700	−20.193	41.245	−4.084	16.561
MFS	−377.216 *	163.948	−4.794	2.586	−4.411	12.438	−7.657	4.814
MLS	−227.299	131.720	1.049	4.981	2.747	18.627	7.640	8.410
MLW	−103.540	428.835	−1.217	27.450	15.815	99.626	52.397	42.627
OMS	192.300	318.730						
RHO	−446.622	367.132						
SKF	−386.916 *	144.517	−2.881	1.791	−6.310	12.242	−9.162	5.699
SUF	6.156	85.791	1.761	1.140	−2.979	6.027	3.372	2.650
TEX	−169.506	116.667	−1.652	1.666	−7.008	9.174	4.096	3.719
WAD	−29.694	174.123						
WBS	−169.948	353.251						
WGH	−1384.466	1051.883						
WHH	−178.101	681.530						
WKF	−83.477	220.721	−5.723 *	2.484	−10.765	18.547	−12.346	12.341

*: *p*-Values < 0.05.

**Table 4:** Across-breed means of the animal model linear regression coefficients of the inbreeding depression derived from the individual rate of inbreeding (ΔF_i_), the ancestral (F_a___Kal_) and new (F_a___New_) inbreeding coefficient according to Kalinowski, inbreeding coefficient (F) and interaction between F and the ancestral inbreeding coefficient according to Ballou (F×F_a_Bal_) for daily weight gain, meatiness score, ultrasound muscle thickness and ultrasound fat thickness with their corresponding standard deviations (SD), standard errors (SE), the 95% and 5% confidence intervals (95% CI, 5% CI) and the *p*-Values. Significant *p*-Values are in bold.

Model			Daily Weight Gain	Meatiness Score	Ultrasound Muscle Thickness	Ultrasound Fat Thickness
Number of breeds			25	12	12	12
3	ΔF_i_	Mean	−146.9863	0.0122	−3.7372	4.1497
		SD	339.1584	4.2035	11.9692	17.5269
		SE	67.8317	1.2135	3.4552	5.0596
		95% CI	231.7485	7.8960	20.8445	52.3974
		5% CI	−446.6217	−5.7226	−20.1934	−12.3457
		*p*-Value	**0.0404**	0.9921	0.3026	0.4295
4	F_a___Kal_	Mean	81.5307	−1.1107	−3.5346	12.2960
		SD	884.5720	4.9456	74.4273	39.3548
		SE	176.9144	1.4277	21.4853	11.3607
		95% CI	1298.9443	9.6708	192.4876	135.1629
		5% CI	−1119.8257	−9.4966	−138.4772	−16.3338
		*p*-Value	0.6491	0.4530	0.8723	0.3023
4	F_a___New_	Mean	−45.7366	−0.2796	−2.2528	−0.6576
		SD	154.9961	1.5670	6.7777	2.9451
		SE	30.9992	0.4524	1.9565	0.8502
		95% CI	163.3335	3.7482	6.8386	4.1681
		5% CI	−374.8156	−2.3767	−19.5289	−6.4020
		*p*-Value	0.1531	0.5491	0.2740	0.4556
5	F	Mean	−28.9572	−0.2056	−1.7407	0.6327
		SD	58.1952	1.0825	4.2869	3.4869
		SE	11.6390	0.3125	1.2375	1.0066
		95% CI	59.0253	1.3965	5.6505	9.9859
		5% CI	−141.7366	−2.1999	−8.7675	−2.5614
		*p*-Value	**0.0202**	0.5241	0.1872	0.5425
5	F×F_a_Bal_	Mean	801.0222	−7.6659	102.9336	21.4346
		SD	4148.0000	23.6919	347.1661	112.0924
		SE	829.6284	6.8393	100.2182	32.3583
		95% CI	3549.2470	34.8358	1182.0997	373.6340
		5% CI	−2919.4690	−64.9516	−121.5639	−47.3431
		*p*-Value	0.3439	0.2862	0.3264	0.5213

**Table 5:** Across-breed means of the animal model linear regression coefficients of the inbreeding depression in percentages derived from the individual rate of inbreeding (ΔF_i_) and scaled to the respective trait mean (ΔF_i_/mean), phenotypic (ΔF_i_/σ_P_) and additive genetic standard deviation (ΔF_i_/σ_A_) and expressed per 1% increase in ΔF_i_ for daily weight gain, meatiness score, ultrasound muscle thickness and ultrasound fat thickness with their corresponding standard deviations (SD), standard errors (SE), the 95% and 5% confidence intervals (95% CI, 5% CI), kurtosis, skewness and *p*-Values. Significant *p*-Values are in bold.

Model			Daily Weight Gain	Meatiness Score	Ultrasound Muscle Thickness	Ultrasound Fat Thickness
Number of breeds			25	12	12	12
3	ΔF_i_/mean	Mean	−0.6121	0.0016	−0.1222	0.7929
		Median	−0.2735	−0.1158	−0.1879	−0.0227
		SD	1.6100	0.5549	0.4002	3.002
		SE	0.3220	0.1602	0.1155	0.8666
		95% CI	0.6823	1.0539	0.6933	9.1162
		5% CI	−1.8128	−0.7502	−0.7338	−1.5407
		Skewness	−3.0729	0.6818	0.8809	2.2538
		Kurtosis	13.4433	−0.0660	0.7707	5.5839
		*p*-Value	0.0694	0.9923	0.3126	0.3798
3	ΔF_i_/σ_P_	Mean	−2.9855	−0.0348	−1.2018	2.7371
		Median	−2.3955	−1.2395	−1.9737	0.1399
		SD	6.6259	6.6269	3.7540	12.7208
		SE	1.3252	1.9130	1.0837	3.6722
		95% CI	4.2630	11.5175	6.0817	38.3887
		5% CI	−11.2270	−10.6266	−7.1350	−7.5415
		Skewness	−1.7125	0.4588	0.8607	2.2837
		Kurtosis	6.5279	−0.2610	0.8097	5.9547
		*p*-Value	**0.0337**	0.9858	0.2911	0.4717
3	ΔF_i_/σ_A_	Mean	−3.9845	−0.1044	−1.3419	4.0202
		Median	−3.2324	−2.5247	−2.7402	−0.0684
		SD	8.0286	11.9353	6.5320	17.8301
		SE	1.6057	3.4454	1.8856	5.1471
		95% CI	5.3994	20.7801	13.9784	55.8558
		5% CI	−14.3202	−19.0753	−11.7174	−9.5718
		Skewness	−1.3538	0.4687	1.1816	2.5572
		Kurtosis	4.6147	−0.2423	2.3159	7.3876
		*p*-Value	**0.0205**	0.9764	0.4915	0.4512

## Data Availability

Restrictions apply to the availability of these data. Data were obtained from vit/Verden and are on a reasonable request available from the authors with the permission of the German sheep breeding associations.

## Supplementary Materials

The following supporting information can be downloaded at: https://www.mdpi.com/article/10.3390/ani13223547/s1, Figure S1. Ultrasound measurement of the Musculus longissimus dorsi (distance between 1 and 2) and the fat layer including the skin above. Table S1. Studies on inbreeding depression in sheep associated with production traits extracted from the meta-analysis by Doekes. Table S2. Number of animals in pedigree file (N), number of complete equivalent generations (GE), individual rate of inbreeding (∆F_i_), inbreeding coefficient for all (F), new (F_a_New_) and ancestral inbreeding coefficient according to Kalinowski, ancestral inbreeding coefficient according to Baumung (AHC) and ancestral inbreeding coefficient according to Ballou (F_a_Bal_). Table S3. Methodology for conducting meat performance testing. Table S4. Estimated heritabilities, additive genetic variances and covariances, as well as residual variances and covariances for the traits of wool quality, muscling conformation, exterior, daily weight gain, meatiness score, ultrasound muscle and fat thickness. Table S5a. Animal model linear regression coefficients of the individual rate of inbreeding (ΔF_i_) on the final score of daily weight gain with their corresponding standard errors (SE) and *p*-Values by breed. Table S5b. Animal model linear regression coefficients of the ancestral (F_a_Kal_) and new (F_a_New_) inbreeding coefficients according to Kalinowski on the final score of daily weight gain with their corresponding standard errors (SE) and *p*-Values by breed. Table S5c. Animal model linear regression coefficients of the inbreeding coefficient (F) and interaction between F and the ancestral inbreeding coefficient according to Ballou (F×F_a_Bal_) on the final score of daily weight gain with their corresponding standard errors (SE) and *p*-Values by breed. Table S5d. Animal model linear regression coefficients of the inbreeding depression derived from the individual rate of inbreeding (ΔF_i_), the ancestral (F_a_Kal_) and new (F_a_New_) inbreeding coefficient according to Kalinowski, inbreeding (F) and interaction between F and the ancestral inbreeding coefficient according to Ballou (F×F_a_Bal_) on the final score of daily weight gain with their corresponding standard deviations (SD), standard errors (SE) and the 95% confidence interval (95% CI), the 5% confidence interval (5% CI) and the *p*-Values for all breeds and the six breeding directions (BD) merino (MER), meat (MEA), country (CON), mountain (MON), heath (HEA) and exotic (EXO). Table S6a. Animal model linear regression coefficients of the individual rate of inbreeding (ΔF_i_) on the final score of meatiness score with their corresponding standard errors (SE) and *p*-Values by breed. Table S6b. Animal model linear regression coefficients of the ancestral (F_a_Kal_) and new (F_a_New_) inbreeding coefficient according to Kalinowski on the final score of meatiness score with their corresponding standard errors (SE) and *p*-Values by breed. Table S6c. Animal model linear regression coefficients between the inbreeding coefficient (F) and interaction between F and the ancestral inbreeding coefficient according to Ballou (F×F_a_Bal_) on the final score of meatiness score with their corresponding standard errors (SE) and *p*-Values by breed. Table S6d. Animal model linear regression coefficients of the inbreeding depression derived from the individual rate of inbreeding (ΔF_i_), the ancestral (F_a_Kal_) and new (F_a_New_) inbreeding coefficient according to Kalinowski, inbreeding (F) and interaction between F and the ancestral inbreeding coefficient according to Ballou (F×F_a_Bal_) on the final score of meatiness score with their corresponding standard deviations (SDs), standard errors (SE) and the 95% confidence interval (95% CI), the 5% confidence interval (5% CI) and the *p*-Values for all breeds and the two breeding directions (BD) merino (MER) and meat (MEA). Table S7a. Animal model linear regression coefficients of the individual rate of inbreeding (ΔF_i_) on the final score of ultrasound muscle thickness with their corresponding standard errors (SE) and *p*-Values by breed. Table S7b. Animal model linear regression coefficients of the ancestral (F_a_Kal_) and new (F_a_New_) inbreeding coefficient according to Kalinowski on the final score of ultrasound muscle thickness with their corresponding standard errors (SE) and *p*-Values by breed. Table S7c. Animal model linear regression coefficients between the inbreeding coefficient (F) and interaction between F and the ancestral inbreeding coefficient according to Ballou (F×F_a_Bal_) on the final score of ultrasound muscle thickness with their corresponding standard errors (SE) and *p*-Values by breed. Table S7d. Animal model linear regression coefficients of the inbreeding depression derived from the individual rate of inbreeding (ΔF_i_), the ancestral (F_a_Kal_) and new (F_a_New_) inbreeding coefficient according to Kalinowski, inbreeding (F) and interaction between F and the ancestral inbreeding coefficient according to Ballou (F×F_a_Bal_) on the final score of ultrasound muscle thickness with their corresponding standard deviations (SD), standard errors (SE) and the 95% confidence interval (95% CI), the 5% confidence interval (5% CI) and the *p*-Values for all breeds and the two breeding directions (BD) merino (MER) and meat (MEA). Table S8a. Animal model linear regression coefficients of the individual rate of inbreeding (ΔF_i_) on the final score of ultrasound fat thickness with their corresponding standard errors (SE) and *p*-Values by breed. Table S8b. Animal model linear regression coefficients of the ancestral (F_a_Kal_) and new (F_a_New_) inbreeding coefficient according to Kalinowski on the final score of ultrasound fat thickness with their corresponding standard errors (SE) and *p*-Values by breed. Table S8c. Animal model linear regression coefficients between the inbreeding coefficient (F) and interaction between F and the ancestral inbreeding coefficient according to Ballou (F×F_a_Bal_) on the final score of ultrasound fat thickness with their corresponding standard errors (SE) and *p*-Values by breed. Table S8d. Animal model linear regression coefficients of the inbreeding depression derived from the individual rate of inbreeding (ΔF_i_), the ancestral (F_a_Kal_) and new (F_a_New_) inbreeding coefficient according to Kalinowski, inbreeding (F) and interaction between F and the ancestral inbreeding coefficient according to Ballou (F×F_a_Bal_) on the final score of ultrasound fat thickness with their corresponding standard deviations (SD), standard errors (SE) and the 95% confidence interval (95% CI), the 5% confidence interval (5% CI) and the *p*-Values for all breeds and the two breeding directions (BD) merino (MER) and meat (MEA). Table S9. Overview of studies in which comparable traits to the meat performance test practiced in Germany were studied by using pedigree data.
