# Supplementary material for: Inbreeding Depression and Purging for Meat Performance Traits in German Sheep Breeds
Source: Animals (Basel). 2023 Nov 17;13(22):3547. doi: 10.3390/ani13223547 (PMC10668769; doi:10.3390/ani13223547)
Supplement: Supplementary file 1 [file animals-13-03547-s001.zip › Figure S1.Method of ultrasound measurements for muscle and fat thickness.pdf]

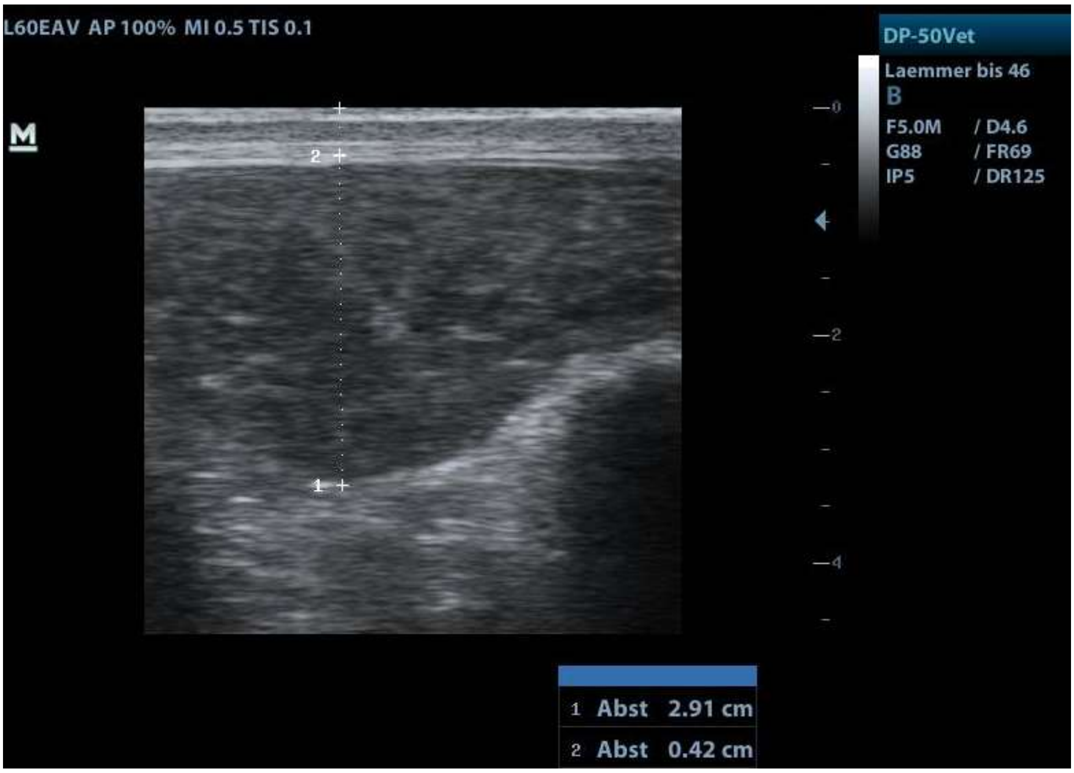

Figure S1. Ultrasound measurement of the Musculus longissimus dorsi (distance between 1 and 2) and the fat layer including the skin above.
