# Supplementary material for: Inbreeding Depression and Purging for Meat Performance Traits in German Sheep Breeds
Source: Animals (Basel). 2023 Nov 17;13(22):3547. doi: 10.3390/ani13223547 (PMC10668769; doi:10.3390/ani13223547)
Supplement: Supplementary file 1 [file animals-13-03547-s001.zip › Table S3.Method of meat performance test.pdf]

Table S3. Methodology for meat performance testing

In order to carry out a breeding value estimation for the trait complex meat performance, various preparations of the data are required. A distinction is made between criteria for commercial breeds and country breeds. The general test criteria, differentiated according to the direction of use of the sheep breed, are essential for the application of the respective estimation models (Table S3a).

Table S3a. General test criteria with reference ranges for economic and country breeds.

Trait	Economic breeds	Country breeds
**Multiple**	Value range 1 – 5	
**Daily weight gain**	150 – 750 g	30 – 500 g
**Ultrasound muscle thickness**	10- 55 mm	-
**Ultrasound fat thickness**	2 – 20 mm	-
**Meatiness score**	1 – 9	-
**Live mass for field inspection**	15 – 100 kg	3 – 80 kg
**Age to field test**	Value range 28 – 210 days	
**Field inspection**	Valid from 1990	
**Field inspection**	Year of acceptance less than or equal to current year	
**Location of field inspection**	Must be known	

In Germany, the meat performance test can take place as a field or station test. The field test takes place on the breeding farm or at events of the breeding association and serves as an own performance test of the lambs whose parents are registered in the herdbook. The test starts directly on the day after birth and ends at the time when the majority of the flock reaches the commercial mast weight typical for the breed. The field test can be carried out on both male and female animals and includes at least the recording of the daily weight gain in gram (g). For the calculation, the birth weight given for the respective breed is subtracted from the test weight and divided by the age in days at the end of the test. Alternatively, the breeder may enter the actual birth weight in the herdbook. In addition, the meatiness grade, muscle and fat thickness can be determined by ultrasound measurement by a representative of the breed association.

To assess the meatiness score, the meat-bearing batches such as breast, shoulder, back and leg are evaluated and weighted according to their economic importance. Values range from 9 – 1 (9 is best).

In addition to the collected measurement data, the following data is also collected for field testing. Test location, identification number with which the animal is registered in the herdbook, as well as the test date and the test weight.

The weight class correction is carried out for the traits ultrasound muscle thickness (USM) and ultrasound fat thickness (USF) within the breeds. According to the 10 classes of approximately

equal number are formed within each breed according to the live mass (LM) for ultrasound measurement (Table S3b).

Table S3b. Classification of the weight classes for the ultrasound readings.
The following procedure is used for each breed with performance observations:

Weight class factor (factor) = (Live mass – Live mass_Mean) / Live mass_StdDev;

The weight classes will be formed as follows:

if		factor	< -1.28	then	Weight class = 1;
if -1.28	<	factor	< -0.84	then	Weight class = 2;
if -0.84	<	factor	< -0.52	then	Weight class = 3;
if -0.52	<	factor	< -0.25	then	Weight class = 4;
if -0.25	<	factor	< 0	then	Weight class = 5;
if 0	<	factor	< 0.25	then	Weight class = 6;
if 0.25	<	factor	< 0.52	then	Weight class = 7;
if 0.52	<	factor	< 0.84	then	Weight class = 8;
if 0.84	<	factor	< 1.28	then	Weight class = 9;
if		factor	> 1.28	then	Weight class = 10;

Ultrasound measurement is a method of collecting data that can be used to infer the composition of the carcass by measuring muscle thickness and fat thickness on the live animal at specified points. In principle, the procedure can be applied to all breeds of sheep of all ages. The weight and date are to be recorded at the same time. An imaging ultrasound measuring device (real-time mode) linear transducer covering a frequency range from 3.5 MHz to 8.0 MHz is required. The transducer used must be specified when recording the measurement data in OviCap.

Method of ultrasound measurement on sheep within the framework of the meat performance test
Muscle thickness is the largest vertical cross-section of the muscle including the muscle fascia. Fat thickness is the sum of solid subcutaneous fat, including the skin over the muscle thickness measurement site.
The measuring point on the sheep where these two parameters are collected is located behind the 13th rib on the right side. Both measurements are taken at this point. Repeated measurements are permitted, the calculated mean value is published. The measured values are given in millimetres with a maximum of one decimal place. The muscle thickness is measured together with the muscle fascia in the image from the lowest point vertically upwards and the fat thickness represents the overlying fat layer including the skin. During the

measurement, the lambs are fixed in a special rack, whereby it is essential to ensure that the animal hangs freely so that the back is relaxed. If no rack is available, the animal is gently pressed against the wall, making sure that the animal stands up straight.
The wool, which is an obstacle to the ultrasound measurement, is parted straight at the measurement site with a thin, pointed object and oil or ultrasound gel is applied to the skin as a contact medium. In order to exclude an influence of the contact medium on the measurement image, an ambient temperature of at least 8 °C should prevail. All ultrasound measurements taken during the performance test have to be carried out by trained personnel.

If the ultrasound measurement is carried out as part of the station test, it is carried out in the 35 - 48 kg section depending on the weight, whereby the target weight is approx. 43 kg. In the field test, the target mast weight is the commercial mast weight specified in the breeding program of the respective breed. The weight on the day of measurement should not vary by more than ± 10 kg around the target mast weight. Field test results between 60 and 210 days of age are used for breeding value estimation.
