# Supplementary material for: Inbreeding Depression and Purging for Meat Performance Traits in German Sheep Breeds
Source: Animals (Basel). 2023 Nov 17;13(22):3547. doi: 10.3390/ani13223547 (PMC10668769; doi:10.3390/ani13223547)
Supplement: Supplementary file 1 [file animals-13-03547-s001.zip › Table S5a-d.AnimalModel_Regression coefficients_DailyWeightGain.pdf]

Table S5a. Animal model regression coefficients of the individual rate of inbreeding ΔF_i on the final score for the daily weight gain with the corresponding standard errors (SE) and *p*-Values within breeds.

Breed	ΔF_i	SE	*p*-Value
AST	-219.376	176.184	0.213
BBS	-158.995	165.840	0.338
BDC	-319.017	240.206	0.184
BLS	539.066	971.206	0.579
BRI	139.508	164.613	0.397
CHA	231.749	190.606	0.224
COF	-37.209	272.417	0.891
DOS	3.339	362.405	0.993
GGH	-382.008	427.587	0.372
IDF	22.494	115.652	0.846
KST	-66.466	212.277	0.754
LES	-69.414	228.218	0.761
MFS	-377.216	163.948	0.021
MLS	-227.299	131.720	0.084
MLW	-103.540	428.835	0.809
OMS	192.300	318.730	0.546
RHO	-446.622	367.132	0.224
SKF	-386.916	144.517	0.007
SUF	6.156	85.791	0.943
TEX	-169.506	116.667	0.146
WAD	-29.694	174.123	0.865
WBS	-169.948	353.251	0.630
WGH	-1384.466	1051.883	0.188
WHH	-178.101	681.530	0.794
WKF	-83.477	220.721	0.705

Table S5b. Animal model regression coefficients of the ancestral (F_{a_Kal}) and new (F_{a_New}) inbreeding coefficient according to Kalinowski on the final score for the daily weight gain with the corresponding standard errors (SE) and *p*-Values within breeds.

Breed	F_{a_Kal}	SE	*p*-Value	F_{a_New}	SE	*p*-Value
AST	-117.162	171.152	0.494	-50.538	80.430	0.530
BBS	45.767	150.202	0.761	-59.303	56.308	0.292
BDC	3583.531	5189.228	0.490	-142.149	99.379	0.153
BLS	419.520	761.240	0.582	47.184	210.310	0.822
BRI	-109.662	368.848	0.766	81.061	77.625	0.296
CHA	-1119.826	428.675	0.009	201.905	104.988	0.054
COF	-138.975	206.807	0.502	9.601	65.702	0.884
DOS	161.285	297.084	0.587	-97.227	143.052	0.497
GGH	-461.009	339.963	0.175	80.143	124.408	0.519
IDF	161.698	307.723	0.599	-0.613	61.071	0.992
KST	-185.687	188.976	0.326	12.191	71.839	0.865
LES	-96.280	182.724	0.598	-3.457	71.242	0.961
MFS	51.919	187.143	0.781	-95.315	45.488	0.036
MLS	26.816	116.228	0.818	-58.689	33.668	0.081
MLW	1298.944	884.991	0.142	-374.816	244.318	0.125
OMS	-502.833	219.949	0.022	163.334	77.052	0.034

**RHO**	-8.865	363.327	0.981	-53.510	114.376	0.640
**SKF**	230.675	197.829	0.244	-101.943	34.623	0.003
**SUF**	-1.528	196.210	0.994	-18.126	30.655	0.554
**TEX**	-140.561	125.249	0.262	-28.309	32.399	0.382
**WAD**	94.974	112.102	0.397	-35.379	70.287	0.615
**WBS**	-218.974	268.862	0.415	40.812	104.094	0.695
**WGH**	391.372	493.349	0.428	-571.160	331.991	0.085
**WHH**	237.148	532.293	0.656	-130.099	248.100	0.600
**WKF**	-94.030	384.676	0.807	-2.071	59.662	0.972

Table S5c. Animal model regression coefficients between the inbreeding coefficient for all (F) and the ancestral inbreeding coefficient according to Ballou (FxF_{a_Bal}) on the final score for the daily weight gain with the corresponding standard errors (SE) and *p*-Values within breeds.

**Breed**	**F**	**SE**	***p*-Value**	**FxF_{a_Bal}**	**SE**	***p*-Value**
**AST**	-68.177	53.042	0.199	-21.797	468.103	0.963
**BBS**	-35.815	38.363	0.351	146.792	363.742	0.687
**BDC**	-141.737	98.886	0.152	19598.959	20624.089	0.342
**BLS**	91.594	136.772	0.503	3549.247	2982.805	0.234
**BRI**	59.025	61.689	0.339	-359.779	824.754	0.663
**CHA**	50.382	91.912	0.584	-2919.469	1067.531	0.006
**COF**	-19.267	40.707	0.636	-667.486	587.264	0.256
**DOS**	-24.950	85.544	0.771	1023.366	982.033	0.297
**GGH**	-52.383	53.061	0.324	82.135	1189.877	0.945
**IDF**	12.060	54.131	0.824	166.090	814.404	0.838
**KST**	-14.652	53.289	0.783	-627.282	476.628	0.188
**LES**	-25.325	41.802	0.545	-195.488	380.666	0.608
**MFS**	-69.853	27.927	0.012	190.409	515.553	0.712
**MLS**	-42.673	21.333	0.045	392.282	308.593	0.204
**MLW**	-33.676	83.311	0.686	1393.421	2490.597	0.576
**OMS**	13.939	44.116	0.752	-613.485	617.099	0.320
**RHO**	-47.614	73.413	0.517	491.001	960.855	0.609
**SKF**	-62.586	22.762	0.006	556.278	461.599	0.228
**SUF**	-15.381	26.724	0.565	153.969	429.747	0.720
**TEX**	-48.158	28.671	0.093	-524.451	416.107	0.208
**WAD**	10.767	41.309	0.794	322.453	264.626	0.223
**WBS**	-25.992	50.314	0.605	-440.059	724.842	0.544
**WGH**	-196.272	151.025	0.194	1777.810	1643.653	0.279
**WHH**	-18.567	84.003	0.825	676.814	1786.228	0.705
**WKF**	-12.285	40.399	0.761	-188.159	932.019	0.840

Table S5d. Animal model linear regression coefficients of the inbreeding depression derived from the individual rate of inbreeding (ΔF_i), the ancestral (F_{a_Kal}) and new (F_{a_New}) inbreeding coefficient according to Kalinowski, inbreeding (F) and interaction between F and the ancestral inbreeding coefficient according to Ballou (FxF_{a_Bal}) on the final score of daily weight gain with their corresponding standard deviations (SD), standard errors (SE) and the 95% confidence interval (95% CI), the 5% confidence interval (5% CI) and the *p*-Values for all breeds and the six breeding directions (BD) merino (MER), meat (MEA), country (CON), mountain (MON), heath (HEA) and exotic (EXO).

	**For all breeds**		**BD**					
			MER	MEA	CON	MON	HEA	EXO
**ΔF_i**	Mean	-140.7631	-236.0180	-53.7375	15.9392	-114.8010	-648.1920	-319.0170

	SD	340.3542	137.0466	191.2472	320.9629	97.8437	645.7319	
	SE	68.0708	79.1239	72.2846	131.0325	48.9218	372.8135	
	95% CI	231.7485	-103.5400	231.7485	539.0661	-3.4153	-178.1010	
	5% CI	-446.6217	-377.2160	-386.9156	-446.6217	-219.3759	-1384.4660	
	p-Value	0.0496	0.0964	0.4853	0.9079	0.1006	0.2242	
	p-Values for differences among BD: p < 0.05 between HEA and MEA, CON, MON							
**F_{a_Kal}**	Mean	81.5307	459.2265	-114.6120	26.7855	-486.5120	55.8366	3583.5310
	SD	884.5720	727.3253	464.7632	210.4285	626.5761	454.1955	
	SE	176.9144	419.9214	175.6640	85.9071	313.2880	262.2299	
	95% CI	1298.9443	1298.9443	230.6749	419.5202	-117.1620	391.3720	
	5% CI	-1119.8257	26.8164	-1119.8257	-138.9746	-1424.2230	-461.0090	
	p-Value	0.6491	0.3883	0.5383	0.7678	0.2183	0.8511	
	p-Values for differences among BD: p < 0.05 between MER and MON							
**F_{a_New}**	Mean	-45.7366	-176.2730	-6.6264	7.5835	-3.4453	-207.0390	-142.1490
	SD	154.9961	172.9153	101.1916	50.3411	39.0928	332.3987	
	SE	30.9992	99.8327	38.2468	20.5516	19.5464	191.9105	
	95% CI	163.3335	-58.6888	201.9052	81.0607	40.8118	80.1433	
	5% CI	-374.8156	-374.8156	-101.9428	-53.5095	-50.5378	-571.1603	
	p-Value	0.1531	0.2195	0.8681	0.7272	0.8713	0.3935	
	p-Values for differences among BD: p < 0.05 between HEA and CON							
**F**	Mean	-68.1960	-110.0409	-16.8187	-63.7743	-2.689	-274.1196	-138.2085
	SD	158.0506	35.2528	115.7149	169.1348	67.6507	282.4362	
	SE	31.6101	20.3532	43.7361	69.0490	33.8254	163.0646	
	95% CI	-374.3650	-88.1415	196.8654	82.4725	68.0904	-68.4067	
	5% CI	96.9226	-150.7072	-178.8932	-374.3650	-65.6724	-596.1377	
	p-Value	0.0412	0.0325	0.7138	0.3981	0.9416	0.2348	
	p-Values for differences among BD: p < 0.05 between MEA and HEA, MON and HEA							
**FxF_{a_Bal}**	Mean	801.0222	658.7038	-247.4824	523.3246	-235.5864	845.5864	19598.959
	SD	4148.0000	644.2402	1278.8691	1543.5152	359.2371	860.3441	
	SE	829.6284	371.9522	483.3671	630.1375	179.6185	496.7199	
	95% CI	3549.2470	1393.421	1023.37	3549.25	146.79	1777.81	
	5% CI	-2919.4690	190.409	-2919.47	-667.49	-627.28	82.13	
	p-Value	0.3439	0.2186	0.6269	0.4441	0.2810	0.2308	
	p-Values for differences among BD: not significant							

Abbreviations for breeding directions: country: CON, exotic: EXO, heath: HEA, meat: MEA, merino: MER, mountain-stone: MON.
