# Supplementary material for: Inbreeding Depression and Purging for Meat Performance Traits in German Sheep Breeds
Source: Animals (Basel). 2023 Nov 17;13(22):3547. doi: 10.3390/ani13223547 (PMC10668769; doi:10.3390/ani13223547)
Supplement: Supplementary file 1 [file animals-13-03547-s001.zip › Table S6a-d.AnimalModel_Regression coefficients_MeatinessScore.pdf]

Table S6a. Animal model regression coefficients of the individual rate of inbreeding ΔF_i on the final score for meatiness with the corresponding standard errors (SE) and *p*-Values within breeds.

Breed	ΔF_i	SE	*p*-Values
BDC	2.332	2.926	0.425
CHA	-2.839	2.335	0.224
DOS	6.766	6.501	0.298
IDF	-0.551	2.426	0.820
LES	7.896	7.700	0.305
MFS	-4.794	2.586	0.064
MLS	1.049	4.981	0.833
MLW	-1.217	27.450	0.965
SKF	-2.881	1.791	0.108
SUF	1.761	1.140	0.122
TEX	-1.652	1.666	0.321
WKF	-5.723	2.484	0.021

Table S6b. Animal model regression coefficients of the ancestral (F_{a_Kal}) and new (F_{a_New}) inbreeding coefficient according to Kalinowski on the final score for meatiness with the corresponding standard errors (SE) and *p*-Values within breeds.

Breed	F_{a_Kal}	SE	*p*-Value	F_{a_New}	SE	*p*-Value
BDC	-5.281	63.527	0.934	0.876	1.179	0.458
CHA	-9.497	6.203	0.126	-1.274	1.308	0.330
DOS	9.671	6.268	0.123	-1.644	2.629	0.532
IDF	-1.835	5.102	0.719	0.063	1.070	0.953
LES	-4.855	4.159	0.243	3.748	1.998	0.061
MFS	-1.142	2.921	0.696	-1.016	0.702	0.148
MLS	1.832	2.932	0.532	-0.371	1.269	0.770
MLW	4.688	48.063	0.922	-2.377	7.611	0.755
SKF	-1.670	2.516	0.507	-0.330	0.434	0.448
SUF	-3.782	2.614	0.148	0.614	0.419	0.143
TEX	-0.214	1.607	0.894	-0.552	0.409	0.177
WKF	-1.243	4.390	0.777	-1.094	0.742	0.140

Table S6c. Animal model regression coefficients between the inbreeding coefficient for all (F) and the ancestral inbreeding coefficient according to Ballou (FxF_{a_Bal}) on the final score for meatiness with the corresponding standard errors (SE) and *p*-Values within breeds.

Breed	F	SE	*p*-Value	FxF_{a_Bal}	SE	*p*-Value
BDC	0.830	1.173	0.479	-64.952	246.690	0.792
CHA	-2.200	1.163	0.058	-17.438	15.272	0.254
DOS	1.326	1.457	0.363	34.836	23.419	0.137
IDF	-0.169	0.913	0.853	-4.498	11.726	0.701
LES	1.397	1.231	0.256	-3.618	11.622	0.756
MFS	-0.984	0.438	0.025	-4.453	8.191	0.587
MLS	0.231	0.741	0.756	5.061	7.605	0.506
MLW	-1.124	4.417	0.799	-30.045	187.508	0.873
SKF	-0.486	0.277	0.079	0.366	5.470	0.947
SUF	0.310	0.366	0.397	-11.105	6.013	0.065
TEX	-0.453	0.384	0.239	5.856	6.528	0.370

**WKF**	-1.145	0.503	0.023	-2.002	10.530	0.849

Table S6d. Animal model linear regression coefficients of the inbreeding depression derived from the individual rate of inbreeding (ΔF_i), the ancestral (F_{a_Kal}) and new (F_{a_New}) inbreeding coefficient according to Kalinowski, inbreeding (F) and interaction between F and the ancestral inbreeding coefficient according to Ballou (FxF_{a_Bal}) on the final score of meatiness score with their corresponding standard deviations (SD), standard errors (SE) and the 95% confidence interval (95% CI), the 5% confidence interval (5% CI) and the *p*-Values for all breeds and the two breeding directions (BD) merino (MER) and meat (MEA).

		For all breeds	BD	
			MER	MEA
**ΔF_i**	Mean	0.0122	-1.6541	-0.7313
	SD	4.2035	2.9461	4.0264
	SE	1.2135	1.7009	1.5218
	95% CI	7.8960	1.0488	6.7655
	5% CI	-5.7226	-4.7944	-5.7226
	p-Value	0.9921	0.4334	0.6479
	p-Values for differences among BD: not significant			
**F_{a_Kal}**	Mean	-1.1107	1.7925	-1.2243
	SD	4.9456	2.9153	5.7048
	SE	1.4277	1.6831	2.1562
	95% CI	9.6708	4.6879	9.6708
	5% CI	-9.4966	-1.1423	-9.4966
	p-Value	0.4530	0.3985	0.5908
	p-Values for differences among BD: not significant			
**F_{a_New}**	Mean	-0.2796	-1.2543	-0.6023
	SD	1.5670	1.0241	0.7934
	SE	0.4524	0.5912	0.2999
	95% CI	3.7482	-0.3708	0.6141
	5% CI	-2.3767	-2.3767	-1.6441
	p-Value	0.5491	0.1679	0.0913
	p-Values for differences among BD: not significant			
**F**	Mean	-0.4037	-0.4203	-0.9132
	SD	1.3852	0.3804	1.4159
	SE	0.3999	0.2196	0.5352
	95% CI	1.8699	0.0187	0.7757
	5% CI	-3.7059	-0.6531	-3.7059
	p-Value	0.3152	0.38042	0.3169
	p-Values for differences among BD: not significant			
**FxF_{a_Bal}**	Mean	-7.6659	-9.8122	0.8594
	SD	23.6919	18.1563	16.8049
	SE	6.8393	10.4825	6.3517
	95% CI	34.8358	5.0612	34.8358
	5% CI	-64.9516	-30.0449	-17.4379
	p-Value	0.2862	0.4481	0.8968
	p-Values for differences among BD: not significant			

Abbreviations for breeding directions: meat: MEA, merino: MER.
