# Supplementary material for: Inbreeding Depression and Purging for Meat Performance Traits in German Sheep Breeds
Source: Animals (Basel). 2023 Nov 17;13(22):3547. doi: 10.3390/ani13223547 (PMC10668769; doi:10.3390/ani13223547)
Supplement: Supplementary file 1 [file animals-13-03547-s001.zip › Table S7a-d.AnimalModel_Regression coefficients_UltrasoundMuscleThickness.pdf]

Table S7a. Animal model regression coefficients of the individual rate of inbreeding ΔF_i on the final score for ultrasound muscle thickness with the corresponding standard errors (SE) and *p*-Values within breeds.

Breed	ΔF_i	SE	*p*-Value
BDC	-15.580	16.799	0.354
CHA	-11.899	16.399	0.468
DOS	20.845	32.880	0.526
IDF	-5.107	6.291	0.417
LES	-20.193	41.245	0.624
MFS	-4.411	12.438	0.723
MLS	2.747	18.627	0.883
MLW	15.815	99.626	0.874
SKF	-6.310	12.242	0.606
SUF	-2.979	6.027	0.621
TEX	-7.008	9.174	0.445
WKF	-10.765	18.547	0.562

Table S7b. Animal model regression coefficients of the ancestral (F_{a_Kal}) and new (F_{a_New}) inbreeding coefficient according to Kalinowski on the final score for ultrasound muscle thickness with the corresponding standard errors (SE) and *p*-Values within breeds.

Breed	F_{a_Kal}	SE	*p*-Value	F_{a_New}	SE	*p*-Value
BDC	-138.477	363.628	0.703	-7.282	6.796	0.284
CHA	-55.651	38.600	0.149	-2.623	8.955	0.770
DOS	5.030	28.972	0.862	5.181	13.613	0.704
IDF	-0.447	22.196	0.984	-4.185	4.077	0.305
LES	-26.911	22.342	0.228	6.839	12.216	0.576
MFS	-0.736	13.760	0.957	-1.189	3.508	0.735
MLS	-6.858	12.423	0.581	1.697	4.468	0.704
MLW	192.488	168.201	0.252	-19.529	28.146	0.488
SKF	-16.557	14.018	0.238	1.593	2.975	0.592
SUF	-16.914	13.625	0.214	-1.464	2.207	0.507
TEX	2.769	9.392	0.768	-1.247	2.444	0.610
WKF	19.848	32.938	0.547	-4.824	5.584	0.388

Table S7c. Animal model regression coefficients between the inbreeding coefficient for all (F) and the ancestral inbreeding coefficient according to Ballou (FxF_{a_Bal}) on the final score for ultrasound muscle thickness with the corresponding standard errors (SE) and *p*-Values within breeds.

Breed	F	SE	*p*-Value	FxF_{a_Bal}	SE	*p*-Value
BDC	-7.693	6.759	0.255	185.294	1420.005	0.896
CHA	-8.768	7.777	0.260	-121.564	96.712	0.209
DOS	5.345	7.354	0.467	56.712	98.021	0.563
IDF	-4.010	3.663	0.274	10.983	58.392	0.851
LES	-3.390	7.126	0.634	-8.047	66.097	0.903
MFS	-1.080	2.072	0.602	-11.461	40.696	0.778
MLS	-0.087	3.010	0.977	-25.250	30.836	0.413
MLW	5.651	15.834	0.721	1182.100	677.336	0.081
SKF	-0.995	1.844	0.589	-39.594	30.969	0.201
SUF	-2.518	1.947	0.196	-17.894	31.478	0.570
TEX	-1.286	2.172	0.554	5.053	31.545	0.873

**WKF**	-2.059	3.749	0.583	18.871	78.786	0.811

Table S7d. Animal model linear regression coefficients of the inbreeding depression derived from the individual rate of inbreeding (ΔF_i), the ancestral (F_{a_Kal}) and new (F_{a_New}) inbreeding coefficient according to Kalinowski, inbreeding (F) and interaction between F and the ancestral inbreeding coefficient according to Ballou (FxF_{a_Bal}) on the final score of ultrasound muscle thickness with their corresponding standard deviations (SD), standard errors (SE) and the 95% confidence interval (95% CI), the 5% confidence interval (5% CI) and the *p*-Values for all breeds and the two breeding directions (BD) merino (MER) and meat (MEA).

		**For all breeds**	**BD**	
			MER	MEA
**ΔF_i**	Mean	-3.7372	4.7172	-3.3178
	SD	11.9692	10.2555	11.0966
	SE	3.4552	5.9210	4.1941
	95% CI	20.8445	15.8148	20.8445
	5% CI	-20.1934	-4.4105	-11.8994
	p-Value	0.3026	0.5092	0.4590
	p-Value for differences among BD: not significant			
**F_{a_Kal}**	Mean	-3.5346	61.6313	-8.8459
	SD	74.4273	113.3662	24.2782
	SE	21.4853	65.4520	9.1763
	95% CI	192.4876	192.4876	19.8482
	5% CI	-138.4772	-6.8576	-55.6508
	p-Value	0.8723	0.4458	0.3723
	p-Value for differences among BD: not significant			
**F_{a_New}**	Mean	-2.2528	-6.3402	-1.0813
	SD	6.7777	11.5126	3.4773
	SE	1.9565	6.6468	1.3143
	95% CI	6.8386	1.6973	5.1808
	5% CI	-19.5289	-19.5289	-4.8235
	p-Value	0.2740	0.4408	0.4421
	p-Value for differences among BD: not significant			
**F**	Mean	-5.9086	-15.7843	-2.0074
	SD	14.4632	30.4658	2.3144
	SE	4.1752	17.5894	0.8748
	95% CI	3.5842	3.5842	2.8414
	5% CI	-50.9010	-50.9010	-4.1875
	p-Value	0.1847	0.4642	0.0615
	p-Value for differences among BD: not significant			
**FxF_{a_Bal}**	Mean	102.9336	381.7963	-12.4904
	SD	347.1661	693.1174	56.7041
	SE	100.2182	400.1715	21.4321
	95% CI	1182.0997	1182.0997	56.7122
	5% CI	-121.5639	-25.2504	-121.5639
	p-Value	0.3264	0.4407	0.5813
	p-Value for differences among BD: not significant			

Abbreviations for breeding directions: country: meat: MEA, merino: MER.
