# Supplementary material for: Inbreeding Depression and Purging for Meat Performance Traits in German Sheep Breeds
Source: Animals (Basel). 2023 Nov 17;13(22):3547. doi: 10.3390/ani13223547 (PMC10668769; doi:10.3390/ani13223547)
Supplement: Supplementary file 1 [file animals-13-03547-s001.zip › Table S8a-d.AnimalModel_Regression coefficients_UltrasoundFatThickness.pdf]

Table S8a. Animal model regression coefficients of the individual rate of inbreeding ΔF_i on the final score for ultrasound fat thickness with the corresponding standard errors (SE) and *p*-Values within breeds.

Breed	ΔF_i	SE	*p*-Value
BDC	-6.048	7.851	0.441
CHA	5.734	7.467	0.442
DOS	19.039	13.786	0.167
IDF	-3.184	2.891	0.271
LES	-4.084	16.561	0.805
MFS	-7.657	4.814	0.112
MLS	7.640	8.410	0.364
MLW	52.397	42.627	0.219
SKF	-9.162	5.699	0.108
SUF	3.372	2.650	0.203
TEX	4.096	3.719	0.271
WKF	-12.346	12.341	0.317

Table S8b. Animal model regression coefficients of the ancestral (F_{a_Kal}) and new (F_{a_New}) inbreeding coefficient according to Kalinowski on the final score for ultrasound fat thickness with the corresponding standard errors (SE) and *p*-Values within breeds.

Breed	F_{a_Kal}	SE	*p*-Value	F_{a_New}	SE	*p*-Value
BDC	12.946	169.883	0.939	-2.389	3.168	0.451
CHA	-16.334	17.129	0.340	3.862	4.030	0.338
DOS	3.475	12.291	0.777	4.168	5.734	0.467
IDF	5.028	9.988	0.615	-2.210	1.874	0.238
LES	-2.949	9.088	0.746	-0.086	4.958	0.986
MFS	-4.042	5.289	0.445	-0.762	1.366	0.577
MLS	2.977	5.606	0.595	0.885	2.008	0.659
MLW	135.163	73.661	0.067	-6.402	12.207	0.600
SKF	2.104	6.544	0.748	-2.269	1.379	0.100
SUF	-2.278	5.918	0.700	0.641	0.971	0.509
TEX	4.982	3.814	0.191	-0.205	0.991	0.836
WKF	6.481	21.701	0.765	-3.124	3.638	0.390

Table S8c. Animal model regression coefficients between the inbreeding coefficient for all (F) and the ancestral inbreeding coefficient according to Ballou (FxF_{a_Bal}) on the final score for ultrasound fat thickness with the corresponding standard errors (SE) and *p*-Values within breeds.

Breed	F	SE	*p*-Value	FxF_{a_Bal}	SE	*p*-Value
BDC	-2.561	3.157	0.417	-22.494	660.275	0.973
CHA	1.464	3.525	0.678	-47.343	42.846	0.269
DOS	3.929	3.073	0.201	-20.644	41.487	0.619
IDF	-1.641	1.682	0.329	19.051	26.276	0.468
LES	-1.004	2.887	0.728	-5.677	27.283	0.835
MFS	-1.330	0.800	0.096	-20.243	15.615	0.195
MLS	1.375	1.360	0.312	-4.309	13.880	0.756
MLW	9.986	6.811	0.143	373.634	301.320	0.215
SKF	-1.634	0.855	0.056	-0.230	14.490	0.987
SUF	0.431	0.855	0.614	-6.169	13.700	0.652
TEX	0.603	0.881	0.494	-9.313	12.761	0.466

**WKF**	-2.026	2.480	0.414	0.953	52.356	0.985

Table S8d. Animal model linear regression coefficients of the inbreeding depression derived from the individual rate of inbreeding (ΔF_i), the ancestral (F_{a_Kal}) and new (F_{a_New}) inbreeding coefficient according to Kalinowski, inbreeding (F) and interaction between F and the ancestral inbreeding coefficient according to Ballou (FxF_{a_Bal}) on the final score of ultrasound fat thickness with their corresponding standard deviations (SD), standard errors (SE) and the 95% confidence interval (95% CI), the 5% confidence interval (5% CI) and the *p*-Values for all breeds and the two breeding directions (BD) merino (MER) and meat (MEA).

		For all breeds	BD	
			MER	MEA
**ΔF_i**	Mean	4.1497	17.4599	1.0784
	SD	17.5269	31.2085	10.5030
	SE	5.0596	18.0182	3.9697
	95% CI	52.3974	52.3974	19.0390
	5% CI	-12.3457	-7.6574	-12.3457
	p-Value	0.4295	0.4348	0.7950
	p-Values for differences among BD: not significant			
**F_{a_Kal}**	Mean	12.2960	44.6992	0.4940
	SD	39.3548	78.4224	7.9464
	SE	11.3607	45.2772	3.0035
	95% CI	135.1629	135.1629	6.4805
	5% CI	-16.3338	-4.0424	-16.3338
	p-Value	0.3023	0.4276	0.8748
	p-Values for differences among BD: not significant			
**F_{a_New}**	Mean	-0.6576	-2.0930	0.1233
	SD	2.9451	3.8216	2.9561
	SE	0.8502	2.2064	1.1173
	95% CI	4.1681	0.8853	4.1681
	5% CI	-6.4020	-6.4020	-3.1243
	p-Value	0.4556	0.4429	0.9158
	p-Values for differences among BD: not significant			
**F**	Mean	-0.0142	-1.6086	1.0153
	SD	3.6547	5.1372	3.5069
	SE	1.0550	2.9659	1.3255
	95% CI	7.1924	1.9600	7.1924
	5% CI	-7.4965	-7.4965	-2.1104
	p-Value	0.9895	0.6419	0.4727
	p-Values for differences among BD: not significant			
**FxF_{a_Bal}**	Mean	21.4346	116.3608	-247.4824
	SD	112.0924	222.9475	1278.8691
	SE	32.3583	128.7188	483.3671
	95% CI	373.6340	373.6340	1023.366
	5% CI	-47.3431	-20.2429	-2919.469
	p-Value	0.5213	0.4614	0.6269
	p-Values for differences among BD: not significant			

Abbreviations for breeding directions: meat: MEA, merino: MER.
